# Individualized Dosimetry to Guide LuTATE Therapy in Pediatric Neuroblastoma

**DOI:** 10.1055/s-0045-1812305

**Published:** 2025-10-09

**Authors:** Kevin London, Justine Trpezanovski, Toby Trahair, Geoff McCowage

**Affiliations:** 1 Department of Nuclear Medicine, The Children's Hospital at Westmead, Sydney Children's Hospitals Network, Westmead, Sydney, NSW, Australia; 2 Discipline of Medical Imaging Science, Sydney School of Health Sciences, Faculty of Medicine and Health, University of Sydney, Sydney, NSW, Australia; 3 Alfred Nuclear Medicine and Ultrasound, Royal Prince Alfred Hospital Medical Centre, Newtown, Sydney, NSW, Australia; 4 Kids Cancer Centre, Sydney Children's Hospital, Sydney Children's Hospitals Network, Randwick, Sydney, NSW, Australia; 5 Children's Cancer Institute, Lowy Cancer Research Centre, University of New South Wales, Sydney, NSW, Australia; 6 Discipline of Pediatrics and Child Health, School of Clinical Medicine, Faculty of Medicine and Health, University of New South Wales, Sydney, NSW, Australia; 7 Department of Oncology, The Children's Hospital at Westmead, Sydney Children's Hospitals Network, Westmead, Sydney, NSW, Australia

**Keywords:** dosimetry, LuTATE, neuroblastoma, pediatric, theranostics

## Abstract

Neuroblastoma is the most common extracranial solid tumor in children. Tumor heterogeneity is a hallmark of neuroblastoma and prognosis can be dismal in the presence of high-risk factors such as N-myc gene amplification, aneuploidy, genetic rearrangement, and age at presentation. I-131 MIBG therapy has traditionally been the radionuclide therapy agent used in pediatric neuroblastoma and is incorporated in many treatment protocols. The high cost and limited availability of I-131 MIBG and the required radiation safety measures are barriers to its widespread use. LuTATE has emerged as a more practical radionuclide therapy agent requiring less stringent radiation safety measures and coupled with GaTATE PET/CT forms a potentially useful theranostic pairing for children with neuroblastoma. LuTATE therapy in adults with neuroendocrine tumors uses an empiric dosing schedule with good results. However, a previous study using empiric dosing of LuTATE in pediatric neuroblastoma was not shown to be effective. We present our use of individualized patient dosimetry to optimize the dose of LuTATE in three children with relapsed/resistant neuroblastoma.

## Introduction

Neuroblastoma is the most common extracranial solid tumor in childhood. 1 2 Prognosis may be poor and radionuclide therapy is often employed in the palliative setting in children with relapsed and/or resistant disease. 3 4 5 Traditionally, I-131 meta-iodobenzylguanidine (I-131 MIBG) has been used as the radiopharmaceutical of choice to treat pediatric neuroblastoma. 6 7 8 Peptide receptor radionuclide therapy (PRRT) using lutetium-177 octreotate (LuTATE) is an effective treatment for adults with neuroendocrine tumors with validated indications and dosing protocols published in established Australasian guidelines. 9 LuTATE PRRT has been studied in children with relapsed neuroblastoma 10 11 ; however, it has not shown evidence of a significant treatment effect when using empirical dosing protocols. 12 This is thought to be due to inadequate tumor radiation delivery at the empiric LuTATE doses administered. These patients often have suppressed bone marrow function and renal impairment related to a high burden of administered chemotherapy agents and other toxic therapies. In such situations, there may be an emphasis on reducing administered LuTATE activity to avoid further hematological and renal toxicity such that tumor radiation delivery may be critically compromised. One strategy to avoid this issue is to use patient-specific dosimetry calculations to maximize tumor dose while keeping radiation dose to critical organs within toxicity tolerance limits. 13 We report our use of individualized patient dosimetry analysis to optimize the dose of LuTATE in three children treated for relapsed/resistant neuroblastoma.

## Patient Selection and LuTATE Administration

Eligibility criteria for LuTATE therapy include sufficient uptake on a GaTATE PET/CT in most of the known tumor burden, with intensity of uptake greater than physiological liver (Krenning score 3). 14 15 Baseline tests are done, including blood testing for electrolytes, urea, creatinine, full blood count and liver function tests, and a nuclear medicine glomerular filtration rate (GFR) calculation. LuTATE is administered as a day-stay procedure in a lead-lined radionuclide therapy room. The patient is infused with 20 mL/kg (maximum 1 L) of an amino acid solution (Synthamin 17 Electrolyte free solution) 16 over 4 hours with the LuTATE administered an hour after commencement of the amino acid infusion and given over 30 minutes. Ondansetron 4 to 8 mg is routinely given prior to the amino acid infusion to mitigate nausea.

Our LuTATE therapy paradigm is to administer the first cycle as a standard dose of 200 to 300 MBq/kg from which dosimetry calculations are performed to estimate kidney radiation dose. The kidney dosimetry is used to guide dose escalation of further cycles of LuTATE to keep below a cumulative renal dose constraint of 23 Gy, similar to a currently recruiting phase II clinical trial investigating LuTATE dose escalation in children with refractory or relapsed high-risk neuroblastoma. 13 Kidney dosimetry calculations are performed on each LuTATE cycle to guide modification of further LuTATE doses. Studies of radiation-associated kidney injury during external beam radiation therapy conclude a similar threshold for limiting renal toxicity. 17 It is acknowledged that numerous confounding variables contribute to the risk of radiation-induced renal damage, such as co-existing renal disease and previous chemotherapy, and patient age at the time of radiation exposure remains an unquantifiable risk factor. In our practice, factors such as bone marrow competency and GFR contribute to subjective adjustment of LuTATE dose to reduce the risk of toxicity.

## Scanner Calibration and Imaging Protocols

Imaging was performed on a GE Discovery NM/CT 870 CZT gamma camera (GE Healthcare). Intrinsic energy maps for Lu-177 were created by the manufacturer using a calibrated Lu-177 source, following standard vendor protocols. Camera sensitivity calibration was similarly performed according to the manufacturer's specifications. Whole-body planar sweeps and focused SPECT/CT (single-photon emission computed tomography/computed tomography) acquisitions of the abdomen were performed at the end of the amino acid infusion (prior to passing urine) and then again at 24 and 48 hours post-therapy to qualitatively evaluate tumoral LuTATE uptake and to perform kidney dosimetry calculations.

For planar imaging, continuous mode sweeps were acquired from vertex to toes with a pixel exposure time of 240 seconds and a pallet velocity of 10 cm/min. For SPECT/CT acquisitions, a step-and-shoot protocol was used with 20 seconds per projection, 128 × 128 matrix, 360° rotation (120 views, 3° per step), and SwiftScan (“acquire during motion between steps”) enabled. CT was performed with an 80 kV tube voltage, Smart mA (current modulation based on noise index) settings (20–120 mA), noise index of 40, and 1.25 mm slice thickness.

SPECT images were reconstructed using ordered subset expectation maximization with four iterations and eight subsets, incorporating CT-based attenuation correction, scatter correction, and proprietary resolution recovery. All images were corrected for scatter and attenuation during reconstruction.

## Dosimetry Method

The post-therapy scans were analyzed using the proprietary GE dosimetry software, “Dosimetry Toolkit” on a Xeleris workstation. An overview of this software is described by Capala et al. 18 For each cycle of LuTATE and on an individual patient-by-patient basis, the 0-, 24-, and 48-hour post-therapy LuTATE scans were loaded and registered to each other. Rigid registration was applied if required for inter-time point alignment, then the kidneys were segmented manually on both the whole-body sweep and SPECT/CT. Accurate LuTATE administered dose and timing of post-therapy scans were entered from our LuTATE therapy worksheet. The calculated kidney residence time (time-integrated activity coefficients) of LuTATE was then entered into MIRDcalc internal dose calculation software. 19 Using the kidneys as the sole source organs, and selecting an appropriate age and sex phantom, MIRDcalc software calculated the estimated kidney absorbed radiation dose in Gy. As mentioned above, a cumulative dose constraint of 23 Gy was used to guide the dose of further LuTATE cycles.

While it is acknowledged that physicist-led dosimetry is the gold standard, our protocol demonstrates a practical and accessible methodology for clinical departments without dedicated physics support. Potential sources of uncertainty, including registration error, organ segmentation variability, and curve fitting assumptions, are recognized and have been minimized through standardized imaging protocols, high-resolution CZT imaging, and quality control steps. Our approach provides departments with appropriate nuclear medicine expertise a pathway for performing clinically useful dosimetry even in the absence of an on-site physicist.

### Case 1

A 6-year-old boy presented with high-risk neuroblastoma arising from an abdominopelvic mass in the right iliac fossa. At diagnosis he had bilateral renal obstruction and mediastinal nodal metastases. Histopathology showed undifferentiated neuroblastoma without N-myc amplification but with complex segmental chromosomal abnormalities (chromosome 19). Initial treatment included induction chemotherapy, busulfan and melphalan consolidation therapy with autologous stem cell, primary tumor resection, involved-field radiation therapy, dinutuximab and 13- *cis* -retinoic acid. Therapy was completed 13 months after diagnosis.

During the subsequent 2 years, the patient experienced two relapses, presenting with recurrent pelvic masses causing ureteric obstruction. Subsequent treatment included chemo-immunotherapy (dinutuximab, irinotecan, and temozolamide 20 ), external beam radiotherapy, and venetoclax combined with chemotherapy. 21 Prolonged myelosuppression and thrombocytopenia required up to 50% reduction in chemotherapy dosing. He was left with small-volume stable abdominal disease and underwent surgical resection to achieve remission.

**Fig. 1:**
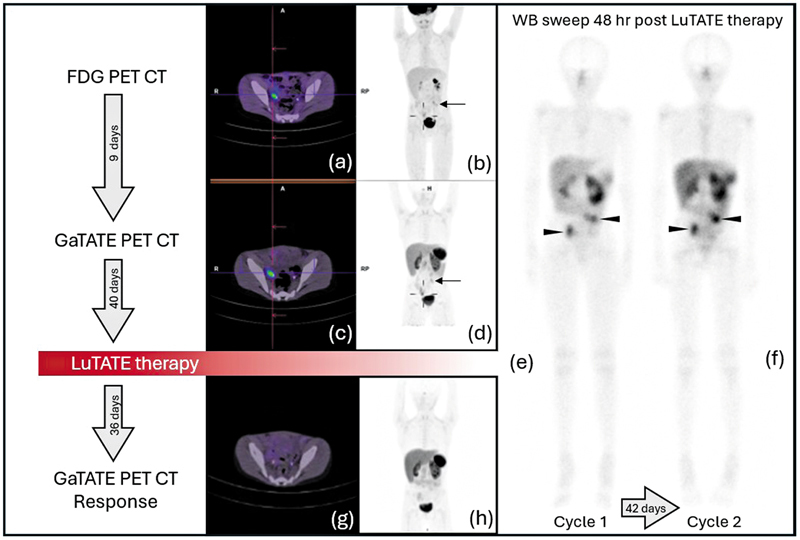
A 9-year-old boy presented with multiple relapsed abdominal high-risk neuroblastoma. Pre-therapy FDG and GaTATE PET/CT, and post-therapy GaTATE PET/CT after the first two cycles of LuTATE are shown. The pre-therapy FDG ( **a** and **b** ) and GaTATE PET/CT ( **c** and **d** ) show uptake with both radiopharmaceuticals in a right pelvic mass (cross hairs) and a left abdominal mass (arrow). Although the masses show significant FDG uptake, the GaTATE uptake is slightly greater than the liver with a Krenning score of 3. The post-LuTATE scans show avid LuTATE accumulation at both masses ( **e** and **f** , closed arrowheads). The response GaTATE PET/CT shows a good partial response with diminished uptake in the abdominopelvic masses ( **g** and **h** ). LuTATE, lutetium-177 octreotate; PET/CT, positron emission tomography/computed tomography.

Three and a half years after diagnosis, a third relapse occurred, presenting with multiple abdominopelvic masses, and he was referred for consideration of radionuclide therapy. FDG and GaTATE PET confirmed variable but generally concordant uptake at two abdominopelvic masses closely associated with the ureters ( Fig. 1 ). MIBG scan was negative (not shown). The patient proceeded to have two cycles of LuTATE. The first cycle was 300 MBq/kg (7.6 GBq) with etoposide sensitization (50 mg/m ^2^ /d orally for 21 days) and dosimetry estimates based on the post-LuTATE imaging showed 7.5 Gy dose to the kidneys. The dose on the second cycle was not escalated due to pre-existing low GFR, resulting from prior chemotherapy and he received another dose of 300 MBq/kg (7.5 GBq) without etoposide sensitization due to myelosuppression. The post-therapy scans showed avid LuTATE accumulation in the abdominopelvic metastases and autologous stem cell reinfusion was delivered after the second cycle. Kidney dose estimate after the second cycle was below dose constraints (5.8 Gy). The response GaTATE PET/CT scan was performed after 36 days and showed a good response with very low-grade uptake in the residual abdominopelvic nodules.

**Fig. 2:**
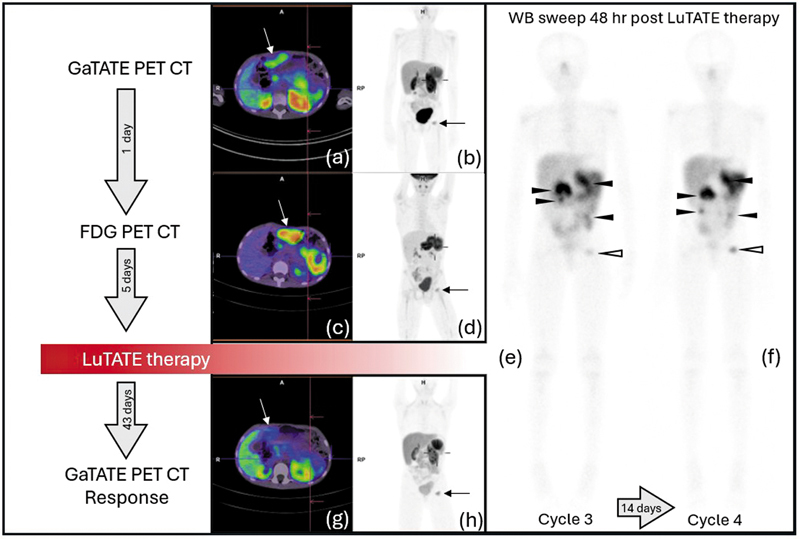
Pre-therapy FDG and GaTATE PET/CT, and post-therapy GaTATE PET/CT after the third and fourth cycles of LuTATE administered to Case 1 are shown. The pre-therapy FDG ( **a** and **b** ) and GaTATE PET/CT ( **c** and **d** ) show uptake with both radiopharmaceuticals in a left abdominal mass (cross hairs), a midline anterior abdominal mass (white arrow), and the left proximal femoral metastasis (black arrow). The recurrent masses continue to show significant FDG uptake and GaTATE uptake greater than the liver with a Krenning score of 3. The post-LuTATE scans show avid LuTATE accumulation in multiple mesenteric masses ( **e** and **f** , closed arrowheads) and the left proximal femoral metastasis (open arrowhead). The response GaTATE PET/CT shows a partial response with diminished uptake in the left abdominal mass ( **g** and **h** , cross hairs) and the midline anterior abdominal mass (white arrow) and stable uptake in the left femoral metastasis (h, black arrow). LuTATE, lutetium-177 octreotate; PET/CT, positron emission tomography/computed tomography.

The next 7 months involved slow disease progression with recrudescence of multiple abdominopelvic masses and eventually a left proximal femoral metastasis visualized on FDG and GaTATE PET/CT ( Fig. 2 ). He then proceeded to have two further cycles of LuTATE with a reduced dose due to low GFR (41–65 mL/min/1.73 m ^2^ ). The third and fourth cycles of LuTATE were 242 MBq/kg (6.1 GBq) and 156 MBq/kg (3.9 GBq), respectively. The post-LuTATE dosimetry estimates confirmed low radiation dose to the kidneys (2.7 and 1.7 Gy, respectively). The response GaTATE PET/CT scan performed 43 days after LuTATE showed partial response with diminished uptake in the abdominopelvic disease and stable uptake in the left proximal femur ( Fig. 2 ).

The subsequent 9 months were characterized by slow disease progression with abdominal, peritoneal, and intra-spinal disease being managed primarily with multiple courses of external beam radiotherapy, and most recently difluoromethylornithine. He remains alive at the time of this report.

### Case 2

**Fig. 3:**
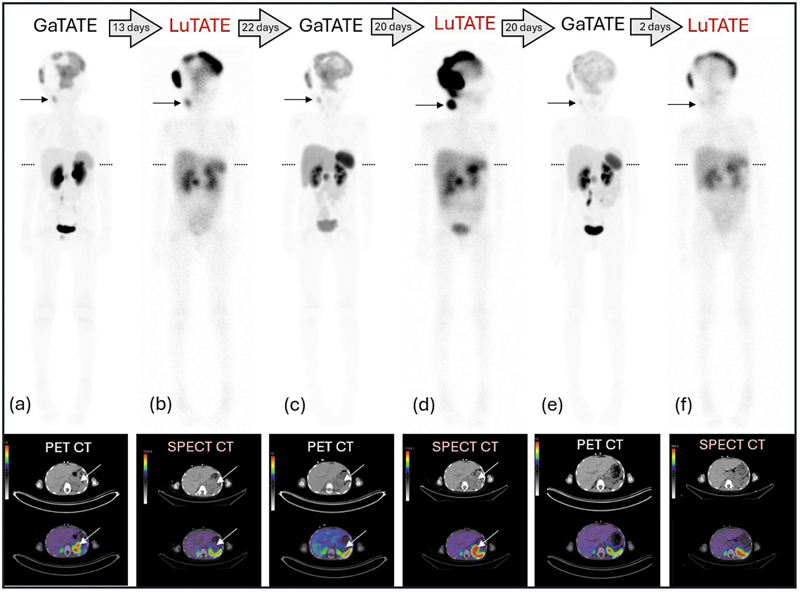
GaTATE PET/CT and 48-hour post-LuTATE scans during three cycles of administered LuTATE to Case 2, a 6-year-old boy with progressive relapsed high-risk neuroblastoma. The GaTATE MIP and 48 hours post-LuTATE WB sweeps are displayed, each with a corresponding axial PET/SPECT CT slice through the upper abdomen (lower panels). The level of the axial slice is indicated on the MIP/WB sweeps (transverse dotted lines). Pre-therapy GaTATE PET/CT ( **a** ) shows extensive avid skull metastases, an avid right cervical nodal metastasis (arrow), and an avid abdominal mass anterior to the left kidney (white arrow). Thirteen days later at the time of the first LuTATE cycle ( **b** ) the skull and right cervical metastases (arrow) show avid LuTATE accumulation, but the abdominal mass does not (white arrow). The absence of LuTATE uptake in the abdominal mass may be explained by interval regression/necrosis of this tumor deposit since the GaTATE PET/CT scan 13 days earlier. The progress GaTATE PET/CT ( **c** ) showed a partial response with unchanged avidity in the skull and right cervical metastases (arrow), but no activity in the abdominal mass (white arrow) like the prior LuTATE post-therapy scan. The second LuTATE dose ( **d** ) administered 20 days later showed very avid uptake in the skull metastases and the right cervical nodal metastases (arrow) and recrudescence of accumulation in the left abdominal mass (white arrow). This implies progression of disease between the GaTATE PET/CT and LuTATE administration. The subsequent GaTATE PET/CT ( **e** ) showed a good partial response with diminished uptake in the skull and right cervical nodal (arrow) metastases, and the left abdominal mass had resolved. This appearance was again shown on the post-therapy scan after the third LuTATE dose ( **f** ). LuTATE, lutetium-177 octreotate; MIP, maximum intensity projection; PET/CT, positron emission tomography/computed tomography; SPECT, single-photon emission computed tomography; WB, whole body.

A 3-year-old boy presented with metastatic high-risk thoracic paraspinal neuroblastoma with nodal, skeletal and marrow metastases, unfavorable histology, and N-myc nonamplified. Initial treatment was with chemotherapy, immunotherapy, and surgical excision of the primary tumor, followed by chemotherapy and stem cell reinfusion. Two and a half years after diagnosis, he recurred in the occipital skull and was treated with surgical excision, chemotherapy, immunotherapy, and external beam radiotherapy. Three months later, he developed an out-of-field recurrence in the inferior occipital skull with multiple smaller skull metastases elsewhere. Treatment included a relapse regimen of chemotherapy and dinutuximab, with subsequent progressive skull, right neck lymph node, and intra-abdominal disease occurring on treatment necessitating external beam radiotherapy. Only the skull metastases were MIBG avid; however, all known sites of disease were GaTATE avid and he was referred for consideration of LuTATE therapy at the age of 6 years. The patient received three cycles of LuTATE and the GaTATE PET/CT scans and 48-hour post-LuTATE scans are shown in Fig. 3 . The pre-therapy GaTATE PET/CT scan showed intense uptake, more intense than liver, in multiple skull metastases, a right cervical nodal metastasis, and a nodal metastasis in the abdomen anterior to the left kidney, showing uptake more intense than physiologic spleen ( Fig. 3a ). The first LuTATE cycle was administered 13 days later (200 MBq/kg (3.6 GBq) with low calculated renal dose (4.2 Gy) and showed avid accumulation in all metastases except the abdominal mass ( Fig. 3b ). The progress GaTATE PET/CT showed a partial response with unchanged avidity in the skull and right cervical metastases, but now no avidity in the abdominal mass ( Fig. 3c ). The second LuTATE cycle was escalated to 422 MBq/kg (7.6 GBq) with renal dosimetry to be within dose constraints (7.3 Gy) and showed avid accumulation in the skull metastases, the right cervical node, and recrudescence of LuTATE avidity in the left-sided abdominal mass ( Fig. 3d ). The following GaTATE PET/CT showed partial response with diminished uptake in the skull and right cervical metastases, and resolution of uptake in the left abdominal mass ( Fig. 3e ). The third LuTATE cycle dose was 478 MBq/kg (8.6 GBq) with LuTATE accumulation in the skull and right cervical nodal metastases only ( Fig. 3f ). Renal dosimetry remained low (5.4 Gy). In the weeks following each LuTATE dose, the numerous bulky skull metastases reduced in size, and after the third cycle, he commenced nivolumab and dinutuximab. Four weeks after the third LuTATE cycle, there was very rapid clinical progression of the skull metastases and significant intracranial extension of the tumors. The patient died of progressive intracranial disease 6 weeks after the third LuTATE cycle.

### Case 3

**Fig. 4:**
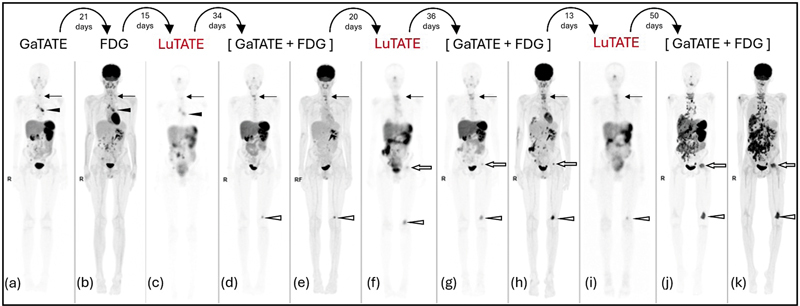
GaTATE PET/CT, FDG PET/CT, and 48-hour post-LuTATE scans during three cycles of LuTATE therapy administered to Case 3, a 15-year-old boy with recurrent high-risk neuroblastoma involving the mediastinal lymph nodes, the cervical spine, and multiple abdominal nodules. The GaTATE and FDG MIP and 48 hours post-LuTATE WB sweeps are displayed in sequence. Prior to the first cycle of LuTATE, there is congruent GaTATE ( **a** ) and FDG ( **b** ) uptake in the cervical spine metastasis (arrow), mediastinal nodal metastases (arrowhead), and multiple smaller foci within the abdomen; and a similar pattern of LuTATE accumulation following therapy ( **c** ). The post-therapy GaTATE ( **d** ) and FDG ( **e** ) PET/CT scans show a mixed response with resolution of the mediastinal and abdominal metastases, and progression of the cervical spinal metastases (arrow) and a new left distal femoral metastasis (open arrowhead). The subsequent second cycle of LuTATE ( **f** ) showed uptake in more osseous lesions, indicating mild disease progression since the GaTATE PET/CT scan. The subsequent GaTATE scan showed stable osseous disease ( **g** ) but the FDG PET/CT showed more extensive FDG uptake at these locations ( **h** ). The emerging discordantly more extensive FDG-avid disease is a worrying sign for dedifferentiated disease progression. The LuTATE uptake on the third cycle ( **i** ) was less prominent than the previous GaTATE with resolution of the GaTATE-positive left femoral head lesions, suggesting a delayed treatment response to previous LuTATE. Fifty days after the fourth LuTATE cycle shows widely progressive GaTATE ( **j** ) and FDG ( **k** ) avid disease. The patient died 23 days later. LuTATE, lutetium-177 octreotate; MIP, maximum intensity projection; PET/CT, positron emission tomography/computed tomography; WB, whole body.

A 15-year-old boy presented with a high-risk large right suprarenal neuroblastoma, non-N-myc amplified with segmental chromosomal abnormalities (chromosomes 1 and 17). Initial treatment was with chemotherapy, partial surgical excision, and stem cell reinfusion. Eight months later, he developed MIBG-negative, GaTATE PET/CT-positive recurrent poorly differentiated neuroblastoma infiltrating the right kidney and commenced dinutuximab and chemotherapy. The patient received three cycles of LuTATE and the GaTATE PET/CT, FDG PET/CT, and 48 hour post-LuTATE scans are shown in Fig. 4 .

Three cycles of LuTATE were administered using escalating doses of 150 MBq/kg (6.6 GBq), 170 MBq/kg (8.2 GBq), and 215 MBq/kg (9.7 GBq), respectively. The dosimetry at all time points showed kidney doses within cumulative dose constraints (3.8, 4.6, and 5.2 Gy, respectively). Prior to the first dose, the pre-therapy GaTATE ( Fig. 4a ) and FDG ( Fig. 4b ) PET/CT scans showed congruent uptake in a cervical spine metastasis (arrow), mediastinal nodal metastases (arrowhead), and multiple smaller foci within the abdomen; and a similar pattern of LuTATE accumulation was seen ( Fig. 4c ). The progress GaTATE ( Fig. 4d ) and FDG ( Fig. 4e ) PET/CT scans showed a mixed response with significant improvement in the mediastinal and abdominal disease, but further congruent avid lesions in the cervical spine (arrow) and left distal femur (open arrowhead). The second cycle of LuTATE ( Fig. 4f ) was given 20 days later and shows mildly more extensive LuTATE accumulation at these locations, with a new focus of accumulation seen in the left femoral head metastasis (open arrow). The subsequent GaTATE scan showed stable uptake ( Fig. 4g ); however, the associated FDG PET/CT scan ( Fig. 4h ) showed mildly more intense and extensive uptake in the cervical spine (arrow), multiple foci in the abdomen, the left femoral head (open arrow), and left distal femur (open arrowhead). The third LuTATE post-therapy scan ( Fig. 4i ) showed less prominent accumulation in the cervical spine (arrow) and left distal femur (open arrowhead), and no uptake in the GaTATE avid left femoral head focus, suggesting a delayed treatment response to previous LuTATE cycles. The subsequent GaTATE ( Fig. 4j ) and FDG ( Fig. 4k ) PET/CT scan showed significant progression of both GaTATE and FDG avid disease at all locations, with extensive and widespread disease in the abdomen and spine. Twenty-three days after the last GaTATE/FDG PET/CT scans, the patient died of extensive, progressive metastatic disease.

Case reports are summarized in Table 1 , including the calculated kidney absorbed radiation dose and hematological toxicity related to the LuTATE therapies.

**Table 1:** The three clinical cases are summarized and presented in table form. Information presented includes patient age at time of first LuTATE cycle, disease status and clinical outcomes following each cycle, GFR prior to each cycle, LuTATE administered activity per cycle, kidney radiation dose per cycle, cumulative kidney dose, and hematologic toxicity for each patient

Case description and age at first LuTATE cycle	Disease status and clinical outcome	GFR (mL/min/1.73 m ^2^ )	LuTATE cycle (days after the previous cycle)	LuTATE dose (GBq)	Kidney dose per cycle (Gy)	Cumulative kidney dose (Gy)	Hematological toxicity
Case 1: 9-year-old male with multiple recurrent neuroblastoma treated with LuTATE 3.5 years after initial diagnosis	Multiple, variably GaTATE avid abdomino-pelvic masses. Less intense FDG uptake. MIBG negative. Administered LuTATE with etoposide sensitization.	101	**1** (first cycle)	7.6 (300 MBq/kg) with etoposide	7.5	7.5	The patient had Grade 1 anemia and thrombocytopenia from previous therapy prior to commencing LuTATE therapy. Following the second LuTATE cycle the patient experienced transient Grade 2 anemia and Grade 4 thrombocytopenia requiring platelet transfusions. Stem cell re-infusion was administered. The anemia and thrombocytopenia both returned to Grade 1 levels prior to the third cycle. Following the fourth cycle the patient developed Grade 3 anemia and Grade 4 thrombocytopenia that continued until the patient's death of progressive metastatic disease.
	Generally stable GaTATE avid disease. Another LuTATE dose given without etoposide due to myelosuppression. Stem cells reinfused after the therapy. Good partial response on GaTATE PET scan.	Not performed	**2** (42)	7.5 (300 MBq/kg), followed by stem cells	5.8	13.3	
	Following an extended period of disease stability, the patient developed progressive GaTATE avid abdomino-pelvic masses and a bone metastasis. LuTATE was given with a reduced dose due to low GFR.	65	**3** (245)	6.1 (242 MBq/kg)	2.7	16.0	
	Fourth cycle given 4 weeks later with a reduced dose of LuTATE due to low GFR. Partial response seen on GaTATE PET scan. Subsequent slowly progressive disease and the patient remains alive 9 months after the last dose of LuTATE.	41	**4** (14)	3.9 (156 MBq/kg)	1.7	17.7	
Case 2: 6-year-old male with multiple recurrent neuroblastoma treated with LuTATE 3 years after initial diagnosis	Extensive MIBG and GaTATE avid skull metastases, right cervical lymph node, and left abdominal metastases. All lesions GaTATE avid, only skull lesions MIBG avid. Partial response on GaTATE PET scan.	144	**1** (first cycle)	3.6 (200 MBq/kg)	4.2	4.2	Prior to commencing LuTATE therapy, the patient had a baseline Grade 2 anemia from previous therapy. Following the LuTATE therapies, there was transient Grade 3 anemia. The patient was not thrombocytopenic prior to LuTATE. Following LuTATE therapy, the patient experienced Grade 3 thrombocytopenia.
	Second LuTATE dose escalated with post-therapy SPECT showing some disease recrudescence. Partial response on GaTATE PET scan.	Not performed	**2** (42)	7.6 (422 MBq/kg)	7.3	11.5	
	Third LuTATE dose escalated further with partial clinical regression of skull metastases. Three weeks after LuTATE developed, rapid skull and intracranial progression and died 5 weeks after the last dose of LuTATE.	139	**3** (22)	8.6 (478 MBq/kg)	5.4	16.9	
Case 3: 18-year-old male with treatment-resistant progressive neuroblastoma.	Extensive FDG and GaTATE avid cervical vertebral, mediastinal nodal and intra-abdominal metastases. Mixed response on GaTATE PET scan.	94	**1** (first cycle)	6.6 (150 MBq/kg)	3.8	3.8	Prior to commencing LuTATE therapy, the patient had Grade 1 anemia from previous therapy, and experienced transient Grade 2 anemia following LuTATE. No other hematologic toxicity occurred.
	Second LuTATE dose escalated. GaTATE PET showed stable disease but slight progression on FDG PET.	86	**2** (54)	8.2 (170 MBq/kg)	4.6	8.4	
	Third LuTATE dose escalated further. Scans performed 50 days after LuTATE showed extensive GaTATE and FDG avid disease progression, and the patient died 73 days after the last dose of LuTATE.	103	**3** (49)	9.7 (215 MBq/kg)	5.2	13.6	

Abbreviations: GFR, glomerular filtration rate; LuTATE, lutetium-177 octreotate; MIBG, meta-iodobenzylguanidine; PET, positron emission tomography; SPECT, single-photon emission computed tomography.

## Discussion

The three cases we have presented all demonstrate the usefulness of individualized dosimetry-guided LuTATE dose prescribing in children with relapsed/recurrent neuroblastoma. Safe dose escalation of LuTATE was achieved in cases 2 and 3 to optimize radiation delivery to the tumors. In case 1, existing chemotherapy-related bone marrow impairment and obstructive kidney dysfunction necessitated no escalation in LuTATE dose during his first two cycles, and in fact, prompted reduced LuTATE doses during his third and fourth cycles of treatment. Despite the reduced LuTATE dosing, this patient remains alive 22 months after his first dose of LuTATE.

Case 2 demonstrates escalating LuTATE doses for three successive cycles, producing a sustained partial response over a period of approximately 4 months. The intra-abdominal metastasis spontaneously lost somatostatin receptor expression between the initial GaTATE PET/CT and the first LuTATE cycle, and the presumed same abdominal mass regained somatostatin receptor expression between the second GaTATE PET/CT and the second LuTATE cycle. This exemplifies the importance of minimizing the time between baseline GaTATE PET/CT imaging and subsequent LuTATE therapy. If these are too distant from each other, unobserved disease progression, or regression, may occur prior to LuTATE administration. Regression of lesions on LuTATE imaging compared with the previous GaTATE PET/CT scan may reflect a delayed treatment response to earlier LuTATE cycles. This phenomenon occurred in case 3 where the third LuTATE cycle showed resolution of the left femoral head metastases ( Fig. 4i ) compared with the immediate prior GaTATE PET/CT scan ( Fig. 4g ). This patient had two previous LuTATE cycles that may be responsible for this. Based on our experience, we recommend patients undergo LuTATE therapy within a week of pre-therapy GaTATE PET/CT assessment.

In case 3, there were also escalating LuTATE doses over three consecutive cycles, producing successive mixed responses, which appeared to hold the overall disease burden at a relatively stable level over a 5-month period. During this time, the patient was able to enjoy an extended overseas holiday with his family, a meaningful positive treatment outcome.

Radionuclide therapy is one of several available options to treat relapsed and refractory neuroblastoma. I-131 MIBG is the most studied theranostic agent for neuroblastoma. Response rates with I-131 MIBG vary widely from 0 to 75% due to the highly heterogeneous patient cohorts studied, imprecise response criteria, and variable administration protocols. 22 I-131 MIBG has been used as monotherapy and in combination with other treatments. 23 24 Most studies have been in early-phase trials in patients with relapsed and refractory high-risk disease. 25 There is one phase 3 trial adding I-131 MIBG therapy to standard induction therapy, which has closed to accrual; however, results are not yet available (ANBL1531, NCT00960739). LuTATE therapy in neuroblastoma is relatively new. Several case series have described its use in neuroblastoma. 11 26 A recently published early-phase clinical trial did not demonstrate significant efficacy at standard empirical doses 12 and the authors acknowledge ineffective empiric dosing of LuTATE may be contributing to the lack of measurable response. Two further clinical trials are underway, both of which incorporate dosimetry calculation and are currently recruiting (NCT04903899 and NCT03966651 13 ).

MIBG and GaTATE have different biodistributions. MIBG is taken up in neuroblastoma cells via the norepinephrine transporter pathway and is highly expressed in over 90% of neuroblastoma patients. LuTATE binds to somatostatin receptors, expressed in up to 85% of patients with neuroblastoma. 11 In addition to differing biodistributions, the different physical properties of the emitted β particles of I-131 MIBG and LuTATE also contribute to their variable effectiveness in patients with neuroblastoma. I-131 emits β particles with an average energy of ∼191 keV compared with 147 keV for Lu-177. 27 Radionuclides emitting lower energy β particles impart their biological effectiveness along a shorter path length than higher energy β particle emitters and Lu-177 has been calculated to have higher efficacy than I-131 against smaller somatostatin receptor-expressing tumors. 28 29 The longer pathlength of the higher energy β particle emitted by I-131 may also impart greater biological effect in tumors with more heterogeneous avidity compared with Lu-177. 25 This may be due to the increased range of the β particle enabling irradiation of the less avid components of the tumor. Other physical factors impact the effectiveness of radionuclide therapy, including half-life and the abundance and energy of emitted gamma photons. Radionuclides emitting α particles and Auger electrons have also been proposed for potential use in patients with neuroblastoma. 25

The optimal role for LuTATE PRRT in view of other treatment options such as molecular targeted therapy 30 and immunotherapy 31 32 33 is yet to be adequately determined. Radiosensitizing agents may also play a role in enhancing the treatment effect of LuTATE in neuroblastoma. 34

For departments to administer effective radionuclide therapy, there is a requirement to minimize the time between PET imaging and delivery of the therapeutic radiopharmaceutical. This is to help prevent unseen rapid disease progression from occurring and thereby disrupting the accurate assessment of response to the therapy in subsequent scans. Particularly where theranostic radiopharmaceuticals are not produced in-house, the careful scheduling of patient scanning appointments, procurement of the required theranostic nuclides, and co-ordination with the patients and their families are important to efficiently provide a theranostics service.

## Conclusion

In conclusion, we have shown the potential for LuTATE PRRT to be an effective radionuclide therapy in the palliation of pediatric patients with relapsed and/or recurrent neuroblastoma. LuTATE is administered as a day-stay procedure with significantly less radiation safety issues compared with I-131 MIBG therapy. Dose escalation of LuTATE, guided by individual dosimetry calculations, forms an important part of LuTATE therapy sequencing, and we have demonstrated how this can be achieved using commercially available software packages in capable nuclear medicine departments that do not have direct support of high-level physics expertise. While similar dosimetric approaches have previously been described with I-131 MIBG therapy 23 35 36 and others have reported their experience in using LuTATE PRRT to treat neuroblastoma, 11 26 our series is the first to report the use of systematic, individualized patient dosimetry to guide dose escalation of LuTATE PRRT in pediatric neuroblastoma. Multicenter prospective trials are required to further optimize the timing and sequencing of LuTATE therapy. LuTATE therapy may be more impactful if used earlier in the disease course of pediatric neuroblastoma.
